# Thermal Transport in Fullerene Derivatives Using Molecular Dynamics Simulations

**DOI:** 10.1038/srep12763

**Published:** 2015-08-04

**Authors:** Liang Chen, Xiaojia Wang, Satish Kumar

**Affiliations:** 1 School of Energy and Power Engineering, Xi’an Jiaotong University, Xi’an, P.R. China; 2 Department of Mechanical Engineering, University of Minnesota, Minneapolis, United States; 3 G. W. Woodruff School of Mechanical Engineering, Georgia Institute of Technology, Atlanta, United States

## Abstract

In order to study the effects of alkyl chain on the thermal properties of fullerene derivatives, we perform molecular dynamics (MD) simulations to predict the thermal conductivity of fullerene (C_60_) and its derivative phenyl-C61-butyric acid methyl ester (PCBM). The results of non-equilibrium MD simulations show a length-dependent thermal conductivity for C_60_ but not for PCBM. The thermal conductivity of C_60,_ obtained from the linear extrapolation of inverse conductivity vs. inverse length curve, is 0.2  W m^−1^ K^−1^ at room temperature, while the thermal conductivity of PCBM saturates at ~0.075  W m^−1^ K^−1^ around 20 nm. The different length-dependence behavior of thermal conductivity indicates that the long-wavelength and low-frequency phonons have large contribution to the thermal conduction in C_60_. The decrease in thermal conductivity of fullerene derivatives can be attributed to the reduction in group velocities, the decrease of the frequency range of acoustic phonons, and the strong scattering of low-frequency phonons with the alkyl chains due to the significant mismatch of vibrational density of states in low frequency regime between buckyball and alkyl chains in PCBM.

Fullerene and its derivatives have gained enormous attention for the application in low-cost, flexible organic photovoltaic devices^1^,^2^,^3^ due to the strong absorption coefficients for ultraviolet and visible light, as the major spectrum of solar radiation^4^,^5^ and relatively higher electron mobility^6^ than other polymers. Besides, owing to the ultra-low thermal conductivity, fullerene and its derivatives have also been studied as advanced thermoelectric materials^7^ and thermal insulation materials for phase change memory devices^8^. A good understanding of the thermal transport mechanism and length dependent thermal properties of fullerene and its derivatives are important in order to accurately consider the effect of Joule heating and heat losses in these devices.

There have been a few experimental studies on the thermal conductivity of fullerene and its derivatives. Thermal conductivity of single crystal C_60_ was first measured using static one-heater, two-thermometer method, and the reported values are around 0.4 W m^−1^ K^−1^ which is nearly temperature independent above 260 K^9^,^10^. Later, 3ω method was used to characterize the thermal conductivity of a C_60_/C_70_ compact, which was shown to be 0.1 W m^−1^ K^−1^ at room temperature^11^. Recently, fullerene derivatives were found to have ultra-low thermal conductivity, which is even lower than that of C_60_ solids. Duda *et al.*^12^,^13^ measured thermal conductivity of phenyl-C61-butyric acid methyl ester (PCBM) using time-domain thermoreflectance (TDTR), and reported exceptionally low values around 0.03 ~ 0.06 W m^−1^ K^−1^ at room temperature. Wang *et al.*^14^ conducted TDTR measurements to study the thermal conductivity of fullerene C_60_ and its derivatives including PCBM and phenyl-C61-butyric acid n-buylester (PCBNB). They observed low thermal conductivity of PCBM and PCBNB around 0.05 ~ 0.06 W m^−1^ K^−1^. In addition, they found the thermal conductivity of disordered C_60_ was about 0.1 W m^−1^ K^−1^ at room temperature, nearly a factor of two larger than that of PCBM or PCBNB. As noted by both groups, the conventional minimum thermal conductivity model^15^ fails to explain the ultralow thermal conductivity of fullerene derivatives by counting the exact atomic number density in a unit cell. Considering two extremes of pure fullerene (disorder C_60_ films)^11^,^14^ and pure organic alkyl-chain systems (amorphous polymers)^16^,^17^, both have higher thermal conductivities than the fullerene derivatives. Therefore, fullerene derivatives such as PCBM and PCBNB cannot be simply treated as a mixture of fullerene and alkyl chains in the study of their thermal conductivity, and their thermal transport mechanisms are complicated by adding the alkyl chains to buckyballs via chemical bonding. Besides, the unusual reduction of thermal conductivity was also observed in the measurements^18^ of the intercalated polymer-PCBM composite. The results showed a lower thermal conductivity of the composite than both of the constituents when the polymer volumetric fraction is below 35%, and similar mechanism was suggested for the reduction of thermal conductivity.

The role of alkyl chains in the thermal transport of fullerene derivatives and their effects on the phonon properties remain unclear. Based on the measurements of picosecond acoustics, both Duda *et al.*^13^ and Wang *et al.*^14^ discovered that the longitudinal speed of sound is ~20%–30% lower in PCBM or PCBNB than that in C_60_. It has been speculated that the alkyl chains attached to buckyballs affect the low frequency phonons, leading to reduction in the speed of sound as well as the thermal conductivity of fullerene. Further study is required for better understanding of the thermal transport mechanisms in fullerene and its derivatives, which is important to engineer their thermal properties with molecular modification and explore the lower limit of thermal conductivity of fully dense solids.

In this work, we study the thermal transport in crystalline C_60_ and its derivative PCBM using molecular dynamics (MD) simulations. We first perform equilibrium MD simulations to obtain the vibrational density of states (VDOS) of crystalline C_60_, disordered C_60_, and crystalline PCBM. We find the mismatch of VDOS in the low frequency regime between buckyballs and alkyl chains in PCBM can lead to strong phonon scattering. We also observe a strong peak near zero frequency in the VDOS of crystalline C_60_ and disordered C_60_ at 300 K, corresponding to the rotation of single buckyball molecule which is totally suppressed in PCBM. Next, we calculate the thermal conductivities of C_60_ and PCBM of different domain lengths (~7 nm to 33 nm) at room temperature using non-equilibrium MD (NEMD) simulations. We observe strong size effects on the thermal conductivity of C_60_. The extrapolated thermal conductivity of C_60_ is 0.2 W m^−1^ K^−1^, which is a factor of 2 larger than that of PCBM (saturates at 0.075 W m^−1^ K^−1^ beyond 20 nm). Based on the different length dependence of the thermal conductivities of C_60_ and PCBM and the reduction in the speed of sound of PCBM observed by the aforementioned works, we attribute the decrease in the thermal conductivity of PCBM to the scattering of low frequency phonons imposed by alkyl chains.

## Results and Discussion

**Figure 1:**
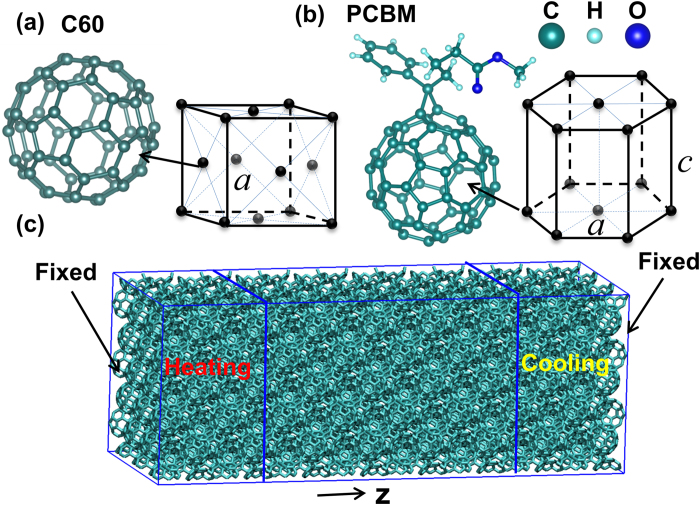
Molecular structures of (**a**) C_60_ and (**b**) PCBM. (**c**) Schematic diagram of system used in NEMD simulations. The system of C_60_ has face-centered-cubic lattice structure with lattice constant of *a* = 13.98 Å. The PCBM has simple hexagonal lattice with lattice constants of *a* = 10.1 Å and *c* = 10.4 Å. Figure drawn by L Chen using VESTA 3.2.1 for the molecular structure.

In MD simulations, the face-centered cubic (FCC) C_60_^19^,^20^,^21^ and simple hexagonal (HEX) PCBM^13^,^22^,^23^,^24^,^25^ are used at room temperature as shown in Fig. 1^43^. The phase transition of C_60_ from simple cubic to FCC occurs at a temperature of 250 K ~ 260 K^19^. But PCBM and other fullerene derivatives have quite different phase behavior due to the pendant groups of alkyl chains attached to C_60_ buckyballs^24^,^25^,^26^. As the annealing temperature increases, PCBM undergoes both amorphous-to-crystalline and crystalline-to-crystalline phase transformations which involve hexagonal crystals and needlelike crystals^24^,^25^. Since previous TDTR measurements indicate that the differences in the thermal conductivities of disordered and microcrystalline PCBM are negligible, so we use HEX lattice for PCBM crystal in the MD simulations.

### Phonon Dispersions of C_60_ and PCBM

**Figure 2:**
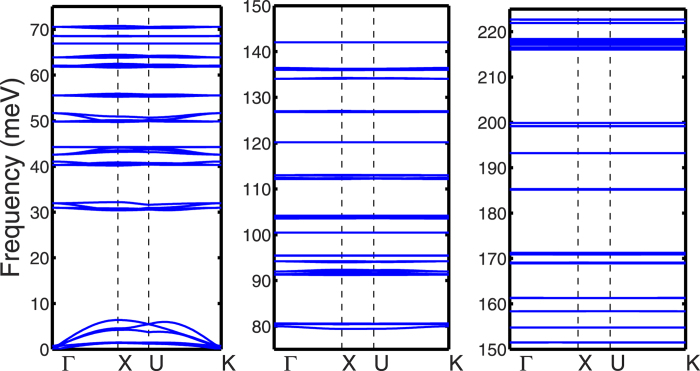
Lattice dynamics calculations of phonon dispersion curves of FCC C_60_. The dispersion curves are divided into three columns of different phonon frequencies for better visualization.

The phonon dispersions of FCC C_60_ crystal and PCBM with HEX lattice are obtained using harmonic lattice dynamics calculations with the inter-atomic force constants (IFCs) determined from frozen phonon method^27^. The primitive unit cell with one molecule is used for both FCC C_60_ and HEX PCBM. The interactions within a 5 × 5 × 5 supercell and a 4 × 4 × 4 supercell are included in the calculation of IFCs for FCC C_60_ and HEX PCBM, respectively. The molecular structures are relaxed in individual lattices using a conjugate gradient energy minimization algorithm at zero temperature. Figure 2 depicts the phonon dispersion curves of FCC C_60_ in its entire frequency range from 0 ~ 225 meV. In order to better elucidate the dispersion features, the frequency range is split into three frequency regimes corresponding to the three columns of Fig. 2. Since the primitive unit cell of 60 carbon atoms is used for the FCC C_60_ crystal, there are 180 branches of dispersion curves. As shown in the first column of Fig. 2, a large gap is observed between the intermolecular phonon branches (below 6.4 meV) and intramolecular phonon branches (above 30 meV). The 174 branches above 30 meV, as illustrated in Fig. 2, correspond to intra-molecular vibrations between bonded carbon atoms, which have negligible contributions to thermal transport due to their small group velocities.

**Figure 3:**
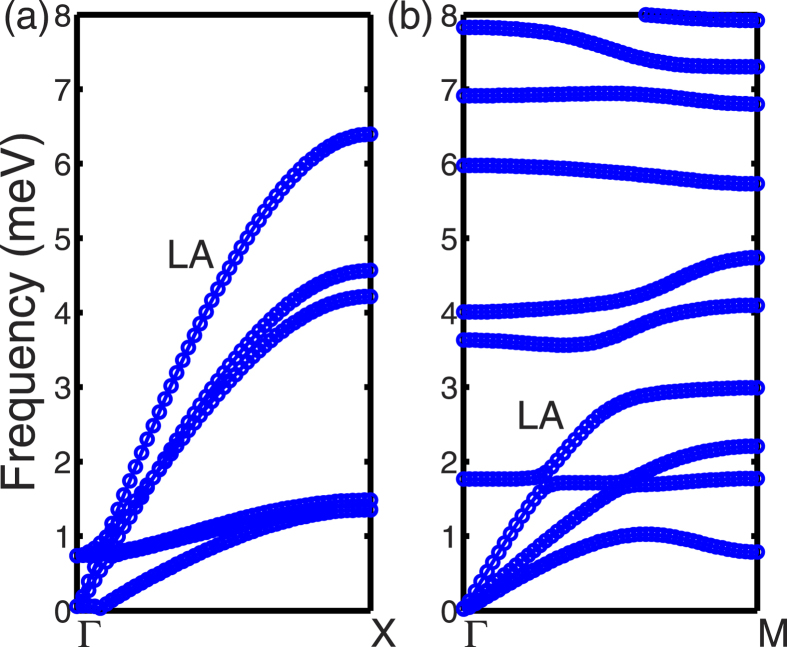
Phonon dispersions at frequency below 8 meV for (**a**) FCC C_60_ and (**b**) simple-hexagonal PCBM.

According to the rigid-molecule approximation^28^, the buckyball in FCC lattice can be treated as a rigid unit with point mass, and its motion is controlled by the net force and the net torque each of which has three degrees of freedom. In our calculations of the FCC C_60_ crystal, there is only one buckyball in each primitive unit cell. Thus there are six branches of dispersion curves below 8 meV, as illustrated in Fig. 3(a), which correspond to intermolecular motions including three translation components and three libration components^21^. In low-frequency regime below 8 meV, three acoustic branches are observed with larger group velocities which characterize the translational vibrations of buckyballs. The other three branches with small group velocity are observed below 1.5 meV which represent the librational vibrations. Here, the librational vibration means the molecules rotate slightly back and forth with a nearly fixed orientation, as restricted by external forces or constraints.

The comparison of dispersion curves between C_60_ and PCBM is shown in Fig. 3 for frequency below 8 meV. It is observed that the maximum frequency of acoustic branches is reduced from 6.4 THz in C_60_ to 3.0 THz in PCBM, which indicates that fullerene is stiffer than its derivatives with alkyl chains. Above the acoustic branches in PCBM, optical branches are observed with small separations. The absence of the band gap in PCMB phonon dispersion between intermolecular modes and intramolecular modes allows more scattering of the low-frequency intermolecular phonons, and thereby lower their phonon mean free path and subsequently the thermal conductivity.

**Figure 4:**
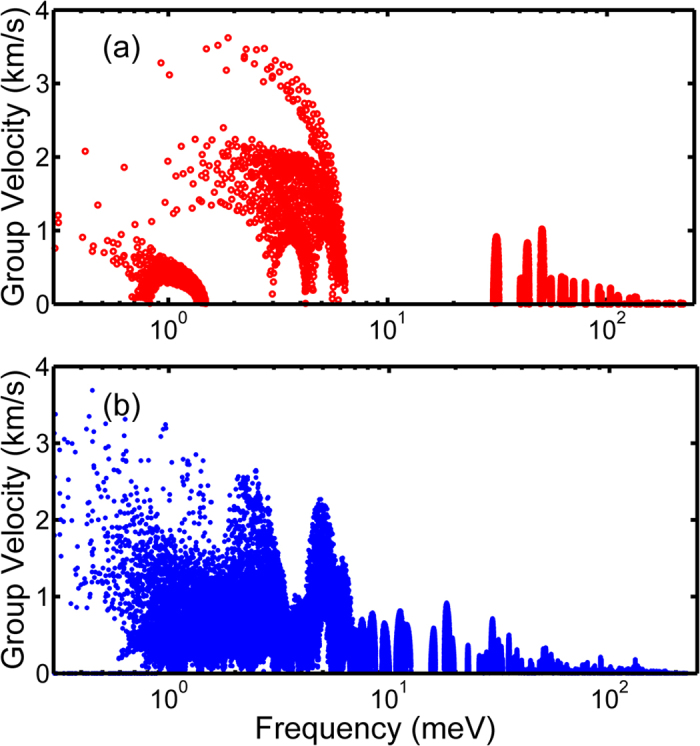
Phonon group velocity as a function of frequency for (**a**) FCC C_60_ and (**b**) simple-hexagonal PCBM.

In Fig. 4, the phonon group velocities as a function of frequency are plotted for FCC C_60_ and HEX PCBM, based on the phonon dispersions determined from LD calculations. The first Brillouin zone (FBZ) is sampled with a 21 × 21 × 21 q-point grid for both FCC C_60_ and HEX PCBM. Figure 4 shows the magnitude of group velocities at the irreducible q-points in the FBZ, e.g., 450 points for FCC C_60_ and 4630 points for HEX PCBM, respectively. The maximum group velocities, corresponding to the longitudinal acoustic branches near Γ point, are ~3.6 km s^−1^ for both FCC C_60_ and HEX PCBM. It can be observed in Fig. 3 that the group velocity (or the slope) of the longitudinal branch in FCC C_60_ remains similar along the Γ-X line, while the group velocity of longitudinal branch in HEX PCBM starts to decrease to zero with the mid-points along the Γ-M line. Moreover, Fig. 4 shows the group velocity in C_60_ has a large population above 1 km s^−1^ while the majority population has group velocities lower than 1 km s^−1^ in PCBM. Therefore, the characteristic group velocity should be lower in PCBM than C_60_. The group velocities shown in Fig. 4 are consistent with the measurement results in literature^12^,^13^,^14^, which has reported the longitudinal speed of sound is ~3.3 ~ 3.8 km s^−1^ in C_60_ and decreases by 20% to 30% in PCBM. It has been suggested that the lower speed of sound in PCBM may be one of the reasons responsible for the reduction in its thermal conductivity compared to C_60_^12^,^13^,^14^,^18^.

### Comparing the Vibrational Density of States

**Figure 5:**
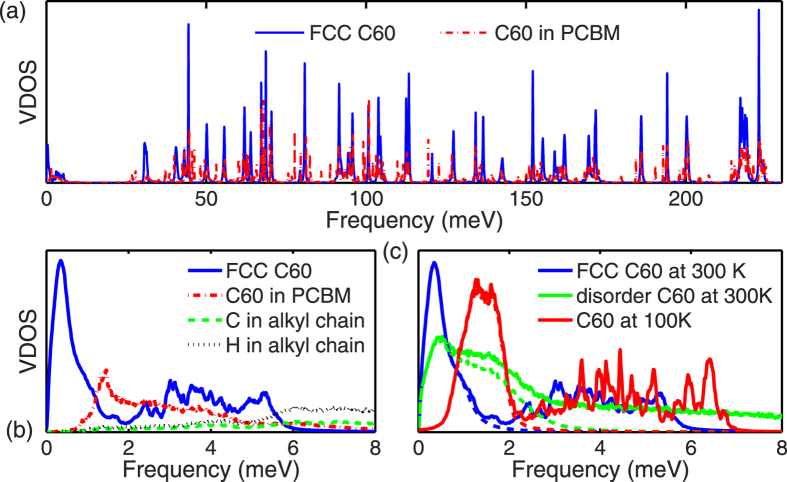
(**a**) VDOS of FCC C_60_, and carbon atoms in simple hexagonal PCBM at 300 K; (**b**) Magnified low-frequency regime of VDOS in Fig. 5(a); also shown in Fig. 5(b) are the VDOS of C and H atoms in the alkyl chain of PCMB. (**c**) Low-frequency regime of VDOS of FCC C_60_ at 100 K and 300 K and disordered C_60_ at 300 K. The dashed lines (partly overlapped with solid lines) indicate the VDOS with translational momentum balanced for each C_60_ molecule.

In order to explore the effects of alkyl chains on phonon states in C_60_ and PCBM, we calculate the VDOS which describes the frequency-dependent distribution of phonon modes. Figure 5(a) plots the VDOS of FCC C_60_ and the buckyball of HEX PCBM at 300 K. In C_60_ or PCBM, the phonon modes with frequency above ~7 meV correspond to the intra-molecular vibrations dominated by the C-C bonding^21^. In Fig. 5(b), a peak centered at 0.35 meV is observed for the VDOS of FCC C_60_ at room temperature, which is also found in previous MD simulations^29^. However, the large population of phonon modes around 0.35 meV is not observed in the dispersion curves shown in Fig. 3(a). The discrepancy is caused by the rotational motion of C_60_ molecules at room temperature. The optimization of C_60_ structure and LD calculations are performed at the temperature of zero Kelvin, and all buckyballs in the supercell have the same orientation as the one in the primitive unit cell. However, the buckyballs of the C_60_ supercell in MD are allowed to rotate; hence the orientation of buckyball can change once the energy barrier between different local minima of energy surface is overcome^30^. At room temperature, the energy barriers to overcome for changing buckyball orientation are negligible compared to their kinetic energy; thus the buckyballs are nearly free to rotate^31^. We speculate that buckyballs in MD simulations undergo continuous diffusive rotational motions^32^ with various orientations, leading to the diminished librational modes.

Neglecting the energy barriers caused by the intermolecular interactions, the isolated buckyball can be treated as the spherical top^32^, a 3D rotator in which all three orthogonal rotations have equal inertia and they are highly symmetric. The DOS of a spherical top has a dependence on the square root of the frequency^33^, which agrees well with the trend of VDOS at near zero frequency for FCC C_60_ at 300 K. Therefore the large VDOS at near zero frequency in C_60_ shall be attributed to the rotational motion of buckyballs. While in PCBM, the VDOS for buckyballs is nearly zero below 1 meV (see Fig. 3(b)) suggesting that the rotational motions are fully suppressed by the alkyl chains.

Compared with the VDOS of FCC C_60_, above 10 meV, the peaks in the partial VDOS of the buckyballs in PCBM are shifted or new peaks are created because of the changes in intra-molecular bonding with the addition of alkyl chains. However, the VDOS of the buckyball in the low frequency regime, dominated by the inter-molecular interaction, have been modified significantly by the alkyl chains in PCBM, as depicted in Fig. 3(b). The VDOS of C and H atoms in alkyl chains of PCMB is also shown in Fig. 3(b). below 5 meV, the VDOS of C and H atoms in alkyl chain is small compared to that of the buckyballs in PCBM. This significant mismatch of VDOS between buckyballs and alkyl chains can cause intense scattering of low frequency phonons. As low frequency inter-molecular phonons may dominate the thermal transport in fullerene, the scattering by alkyl chains can lead to significant decrease of thermal conductivity.

In order to gain some insight on the phonon modes contributing the VDOS peak near zero frequency in C_60_, we compare the VDOS of FCC C_60_ solid at 300 K and 100 K, and disordered C_60_ at 300 K in Fig. 3(c). Also shown in Fig. 3(c) are the VDOS plots (dashed lines) with translational momentum balanced for each C_60_ molecule. Here the translational momentum is balanced by removing the overall translational velocity components of a buckyball from the velocities of each atom in that buckyball^34^. It can be seen from Fig. 3(c), the removal of the translational momentum for each C_60_ molecule does not affect this low-frequency peak (<2 meV), but results in suppression of the VDOS beyond 2 meV. It should be noted that the similar phenomenon has been observed previously^19^. In disordered C_60_, the VDOS peak near zero frequency is broadened to 4 meV, beyond which the VDOS diminishes to zero with the removal of translational momentum.

As the temperature reduces to 100 K, the rotation of buckyball is significantly hindered or even completely frozen near zero frequency for C_60_ at 100 K, as shown in Fig. 3(c). The peak of VDOS at around 1.5 meV for C_60_ at 100 K is not affected with the removal of the translational momentum. The suppression of VDOS at close-to zero frequencies (<0.5 meV) at 100 K can be attributed to the freezing of low frequency rotational motions of the buckyballs, as at low temperatures the kinetic energy is not high enough to overcome the energy barrier between local minima. The MD calculations of the VDOS agree well with the LD predictions for both the FCC C_60_ and the HEX PCBM at low temperatures.

**Figure 6:**
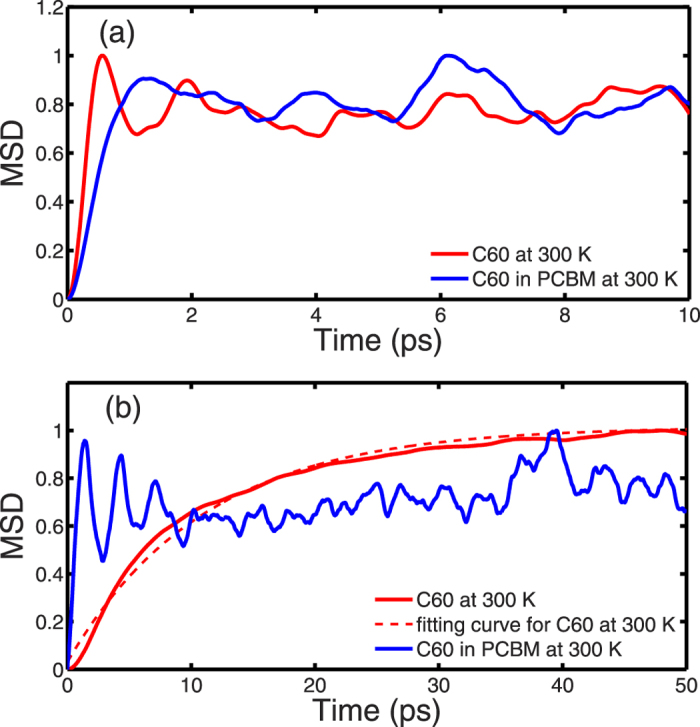
The normalized MSD as a function of time calculated for (**a**) mass center translations of molecules; (**b**) atom displacement after the mass center translation is removed. The MSDs are normalized to their maximum values during the period shown in the figure. The MSD of mass center of translation is 0.14 Å^2^ for both C_60_ and PCBM at 300 K in Fig. 6(a). MSD of rotation is 23.6 Å^2^ for C_60_ and 0.26 Å^2^ for PCBM at 300 K in Fig. 6(b).

To confirm our hypothesis on the correlation of the first low-frequency peak around 0.35 meV in the VDOS of C_60_ at 300 K and the buckyball rotational motion, we calculated the mean square displacement (MSD) which measures the spatial extent of random motion. In this work, the MSD is calculated in two ways: 1) the MSD of the mass center of the molecules, and 2) the MSD of atoms with the mass center translation removed. Figure 6 depicts the MSD as a function of time for C_60_ and PCBM. The MSD in Fig. 6 is normalized to its maximal value in the plotted range of time. It can be seen from Fig. 6(a) that the MSD of the mass center of molecules converges quickly to a small value (~0.14 Å^2^) within 1 ps for both C_60_ and PCBM which indicated no drift of the structure during the simulation.

The MSD of the atoms with the mass center translation removed reflects the spatial extent of the rotational motion. As shown in Fig. 6(b), the profile of MSD of rotation in PCBM is similar to that of the MSD of molecule translation: it reaches the magnitude of 0.26 Å^2^ within 1 ps and then fluctuates. However, the MSD of rotation in C_60_ is much higher (~23.6 Å^2^), and it converges to the maximum value slowly. The profile of MSD versus time for C_60_ in Fig. 6(b) is fitted to a response function, 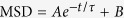. A time constant of 11 ps is obtained. This time constant gives a frequency of 0.37 meV which is in good agreement with the frequency (~0.35 meV) of the VDOS peak in Fig. 5(b) for C_60_ at 300 K. Therefore, the low frequency peak in VDOS for C_60_ at 300 K is attributed to the rotation of C_60_ molecules.

### Thermal Conductivity Reduction in Fullerene Derivatives

**Figure 7:**
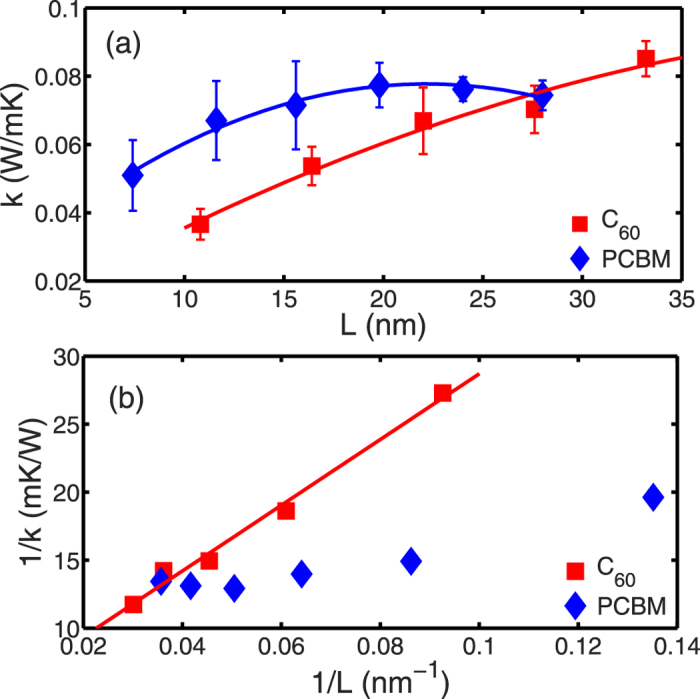
(**a**) Thermal conductivity of FCC C_60_ or simple hexagonal PCBM as a function of simulation domain size *L*. (**b**) Inverse of length-dependent thermal conductivity and the linear fitting of *1/k ~ 1/L* for FCC C_60_. The error bars in figure (**a**) denote the standard deviations calculated from MD samples.

The thermal conductivities of FCC C_60_ and HEX PCBM are calculated using NEMD simulations^35^. In the NEMD simulations, size effects should be cautiously examined due to several reasons^36^,^37^: 1) the phonon mean free path may be larger than the system size; 2) long wavelength phonon modes are absent in the small system; 3) temperature jump at the boundary of heating/cooling bath. The NEMD predictions of thermal conductivity are shown in Fig. 7 for FCC C_60_ and HEX PCBM with different length along the *z* direction of the simulation domain. It can be observed that the thermal conductivity of FCC C_60_ increases as the length increased to 33 nm. But the thermal conductivity of PCBM saturates at ~0.075 W m^−1^ K^−1^ beyond 20 nm. To compensate the contributions to the thermal conductivity of FCC C_60_ from phonons with mean free path longer than 33 nm, we derive a linear relationship between 1/*k* and 1/*L* as shown in Fig. 4(b), and use the linearly extrapolated thermal conductivity of 0.2 W m^−1^ K^−1^ as the model prediction for FCC C_60_.

Our NEMD predictions are smaller than the measured values of ~0.4 W m^−1^ K^−1^ around room temperature by Yu *et al.*^9^ The discrepancy may be caused by the uncertainty in the measurements and the error in linear fitting in a relatively small range of system size considered in our NEMD simulations^36^. Besides, the difference in mass density of C_60_ can also yield quite different thermal conductivity which has been already demonstrated in a previous study^38^.

The predictions of FCC C_60_ are larger than the previously reported thermal conductivity of ~0.1 W m^−1^ K^−1^ for highly disordered C_60_ film from TDTR measurements by Wang *et al.*^14^, and the C_60_/C_70_ compact from 3ω measurements by Olson *et al.*^11^. This indicates the importance to include the factors limiting the phonon mean free path in the measured samples: 1) the C_60_ molecules were highly disordered; 2) grain boundary presented; 3) impurities and defects could not be avoided. Our NEMD prediction of thermal conductivity of HEX PCBM is also higher than the measurements by Wang *et al.*^14^ We attribute the discrepancy between the model prediction and the measured values for PCBM to the grain boundary, impurities, defects, the errors induced by the force field, and the uncertainties of the MD simulations.

Comparing the thermal conductivity predictions for FCC C_60_ and PCBM, we observe a decrease of ~63%. This suggests the increased scattering by the alkyl chains for the dominant phonon modes in C_60_. As shown in Fig. 2 for the phonon dispersion curves of FCC C_60_, the high-frequency intra-molecular phonon modes are highly localized with very small group velocities; therefore, they contribute less to the thermal transport as compared with the low frequency inter-molecular phonons. Moreover, the comparison of VDOS shown in Fig. 5(b) also indicates strong scattering of low frequency phonons of buckyballs by the alkyl chains due to mismatch of phonon distribution. The low frequency phonons mostly consist of inter-molecular modes including both translational modes and librational modes. The individual contributions to thermal transport from the translational and librational modes are not yet determined. Further separation of the individual contributions to the suppression of the thermal conductivity of fullerene derivatives, from either librational or translational modes, requires a more detailed and systematic investigation of the mode-dependent phonon lifetime.

## Conclusions

In summary, we have studied the VDOS and length-dependent thermal conductivity of crystalline C_60_ and PCBM solids using MD simulations. We find there exists a significant mismatch of vibrational density of states in low frequency regime between buckyball and alkyl chains in PCBM. We also observe a larger phonon population with smaller group velocities at low frequencies in PCBM than C_60_. Based on the NEMD predictions of length-dependent thermal conductivity, we obtain an extrapolated value of 0.2 W m^−1^ K^−1^ for FCC C_60_ and a saturated value around 0.075 W m^−1^ K^−1^ for PCBM. The observed 62% reduction in the thermal conductivity from FCC C_60_ to PCBM can be attributed to the reduction in group velocities and the frequencies of acoustic phonons, and the increased scattering of low frequency phonons by the alkyl chains due to mismatch of phonon spectrum.

## Methods

### Molecular Dynamics Simulation Parameters

We use Lammps package^39^ for MD simulations and polymer consistent force field (PCFF) to describe the inter-atomic interactions including van der Waals, bonding, bond-bond angle, dihedral, and improper interactions^40^,^41^. The lattice constant of FCC C_60_ is *a* = 13.98 Å which is determined by matching the measured mass density (1.75 g cm^−3^) of C_60_ samples^14^. For HEX PCBM, due to the difficulty in direct positioning of the PCBM molecule at each lattice site with optimized lattice constants, we first generate the initial structure with a large lattice constant, and then relax the system in isothermal-isobaric (NPT) ensembles. The optimized lattice constants of HEX PCBM are *a* = 10.1 Å and *c* = 10.4 Å, yielding a mass density of 1.65 g cm^−3^ which is within the range of density (1.4 ~ 1.8 g cm^−3^) of PCBM samples in previous experiments^14^,^26^. For the calculation of VDOS, periodic boundary conditions are applied in all three Cartesian directions, and a supercell of 5.6 nm × 5.6 nm × 5.6 nm is used for C_60_ while the domain size of PCBM is 5 nm × 5 nm × 5 nm. In the NEMD simulations, periodic boundary conditions are applied in the *x* and *y* directions while the two ends in the *z* direction are fixed as shown in Fig.1(c). A time step of 0.25 fs is used in all MD simulations.

### Non-equilibrium Molecular Dynamics Simulations

A heating bath and a cooling bath are applied at left end and right end of the system, respectively. The heating/cooling bath is 3 nm long with ~4500 atoms. The constant heat transfer rate 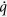 at +/−2 nW is maintained in heating/cooling bath by rescaling the velocities of atoms. In order to reach the steady state, we first perform NEMD simulations for 5 ns before sampling. Then we sample the temperature along the *z* direction every 1.25 ns for another 12.5 ns. According to Fourier’s law, the thermal conductivity is calculated as 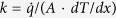, where *A* and *dT*/*dx* are cross-section area and temperature gradient in heat flux direction. The linear fitting of temperature profiles and calculation of thermal conductivity are performed every 1.25 ns. The reported thermal conductivity is the average of 10 samples for the period of 12.5 ns.

### Vibrational Density of States and Mean Square Displacement

The VDOS g(*ω*) is calculatedfrom the sampling of atom velocities in equilibrium MD simulations at room temperature^42^:
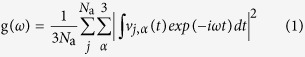
where *N*_*a*_ is the number of atoms, and *v*_*j,α*_ is the velocity of atom *j* in direction α. The mean square displacement (MSD) of the mass center translation is given by
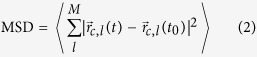
where 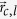 is the position of the mass center of the molecule *l*, and the summation is over all the *M* molecules. The angled brackets denote an ensemble average. The MSD of atoms with the mass center translation is removed is given by
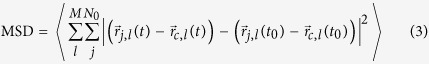
where 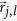 is the position of the atom *j* of molecule *l*, and the summation is performed over all the *N*_0_ atoms in a molecule and then over all the *M* molecules. The positions and velocities are sampled every 20 steps for 2,000,000 steps. In PCBM, only the velocities of carbon atoms of the buckyball are sampled.

## Additional Information

**How to cite this article**: Chen, L. *et al.* Thermal Transport in Fullerene Derivatives Using Molecular Dynamics Simulations. *Sci. Rep.* **5**, 12763; doi: 10.1038/srep12763 (2015).
